# Mechanistic insights into voriconazole-exacerbated selpercatinib-induced thrombocytopenia: a pharmacokinetic interaction case report

**DOI:** 10.3389/fonc.2026.1680060

**Published:** 2026-03-19

**Authors:** Haochun Tang, Ziyuan Zhou, Yuelin Zhang, Wenjun Chen, Jun Meng, Guohui Li

**Affiliations:** 1 Department of Pharmacy, National Cancer Center/National Clinical Research Center for Cancer/Cancer Hospital and Shenzhen Hospital, Chinese Academy of Medical Sciences and Peking Union Medical College, Shenzhen, China; 2 School of Pharmacy, Guangdong Medical University, Dongguan, Guangdong, China

**Keywords:** cancer, CYP3A4 inhibitor, selpercatinib, thrombocytopenia, voriconazole

## Abstract

This paper reports a case of Grade IV thrombocytopenia in a 65-year-old male patient with advanced lung cancer after 4 days of concurrent treatment with selpercatinib and voriconazole. The clinical pharmacist attributed the thrombocytopenia to voriconazole exacerbating the hematological toxicity of selpercatinib and thus stopped selpercatinib and voriconazole treatment sequentially; the patient was given recombinant human thrombopoietin to raise platelets. When the platelet count returned to normal and voriconazole was discontinued, selpercatinib was restarted at a reduced dose and gradually titrated back to the original therapeutic dose. Subsequent treatment did not have any further adverse reactions. This case suggests that doctors and clinician pharmacists should be aware of the potential interaction between selpercatinib and voriconazole, avoid the use of CYP3A4 inhibitors during selpercatinib treatment, and pay attention to dose adjustment.

## 1 Introduction

Transfection rearrangement (RET) is a proto-oncogene, and RET alterations are found in a variety of cancers, including non-small cell lung cancer (NSCLC) and thyroid cancer. Selpercatinib is a selective RET inhibitor that has been approved for the treatment of adult patients with locally advanced or metastatic NSCLC who are positive for RET gene fusions (1). Selpercatinib is mainly metabolized by the cytochrome P450 enzyme system, which is dominated by the CYP3A4 enzyme system. Voriconazole, a potent inhibitor of the CYP3A4 enzyme system, when used in combination with selpercatinib, may lead to decreased metabolism and increased plasma exposure of selpercatinib, thereby increasing the incidence and severity of its adverse effects. In this paper, we report a case of Grade IV thrombocytopenia in a 65-year-old male patient with advanced lung cancer who was receiving concurrent selpercatinib and voriconazole. The aim is to explore the mechanism of selpercatinib-induced thrombocytopenia and observe the drug–drug interaction between selpercatinib and CYP3A4 inhibitors like voriconazole, providing guidance for the rational clinical use of these drugs.

## 2 Case information

### 2.1 Patient history

The patient was a 65-year-old man with a height of 173 cm and a weight of 62 kg. Past history, allergic history, personal history, and family history were unremarkable. The patient was diagnosed with advanced NSCLC with positive RET gene fusion and brain metastasis in November 2024. He started selpercatinib capsule [Manufacturer: Lilly del Caribe, Inc. Batch No.: D842363A] 160 mg twice daily (bid) monotherapy on 15 November 2024. Regular follow-up blood tests during this period showed no significant abnormalities.

### 2.2 Clinical course

On 6 January 2025, the patient was admitted to our hospital with suspected oral *Candida* infection. Admission physical examination: T 36.5°C, P 67 beats/min, and BP 123/86 mmHg; the patient was in good spirits, breathing steadily, with no jaundice or petechiae on the skin or mucosa. Neck was supple, jugular veins were not filled, breath sounds were coarse bilaterally with no dry or wet rales, heart rhythm was regular with no murmurs, the abdomen was flat and soft with no tenderness or rebound pain, and the liver and spleen were not palpable subcostally. There was no edema in the two lower limbs, and the muscle tone in the four limbs is roughly normal.

### 2.3 Laboratory examination:

WBC 4.51×10^9^·L^−1^, N 2.82×10^9^·L^−1^, PLT 125×10^9^·L^−1^, ALT 10 U·L^−1^, AST 11 U·L^−1^, and bilirubin 4.1 μmol·L^−1^. White curd-like pseudomembrane was observed on the oral mucosa. Following pathogenic culture, empirical antifungal treatment with oral voriconazole tablets (Manufacturer: Beijing Bokangjian Genetic Science and Technology Co., Ltd., Batch No. 20240348) was started on 6 January with a loading dose of 400 mg every 12 h (q12h), followed by a maintenance dose of 200 mg q12h from the second day onward. Because voriconazole is a CYP3A4 inhibitor, according to the drug specification of selpercatinib, the dose of selpercatinib needs to be lowered when selpercatinib is used in combination with a CYP3A4 inhibitor, so the patient’s dosage of selpercatinib was adjusted downward to 80 mg, bid, starting from 6 January. The cotton swab culture method returned *Candida* culture (+) on 10 January. Admission diagnosis: advanced NSCLC, positive RET gene fusion, brain metastasis, and oral *Candida* infection.

On 8 January, after 2 days of concurrent treatment with selpercatinib and voriconazole, blood routine was checked: PLT 76×10^9^·L^−1^, indicating Grade I thrombocytopenia. On 10 January, blood routine was rechecked: PLT 23 × 10^9^·L^−1^, indicating Grade IV thrombocytopenia; other blood cell counts were normal, and the patient did not have bleeding symptoms. The physician attributed the thrombocytopenia to selpercatinib, so he suspended selpercatinib and gave recombinant human thrombopoietin injection 15,000 U subcutaneously once daily (qd). 13 January routine blood: PLT 65×10^9^·L^−1^; 16 January routine blood: PLT 108×10^9^·L^−1^, leading to discontinuation of recombinant human thrombopoietin. On 19 January, PLT 112×10^9^·L^−1^, the oral leukoplakia had resolved, suggesting improvement of *Candida* infection, and voriconazole was discontinued. 23 January routine blood: PLT 123×10^9^·L^−1^, so the treatment of selpercatinib 80 mg, bid was restarted. 26 January routine blood: PLT 130×10^9^·L^−1^. 29 January routine blood: PLT 137×10^9^·L^−1^. 30 January: selpercatinib dose was adjusted to 120 mg, bid. 2 February blood: PLT 119×10^9^·L^−1^. 5 February blood: PLT 126×10^9^·L^−1^. 6 February: selpercatinib dose was adjusted to 160 mg, bid. 9 February blood: PLT 131×10^9^·L^−1^. The patient’s blood counts were routinely monitored during the restart of selpercatinib treatment and the gradual upward adjustment of the dose, and the PLT level was maintained at 120–140 ×10^9^·L^−1^, with no other abnormalities noted.

**Figure 1:**
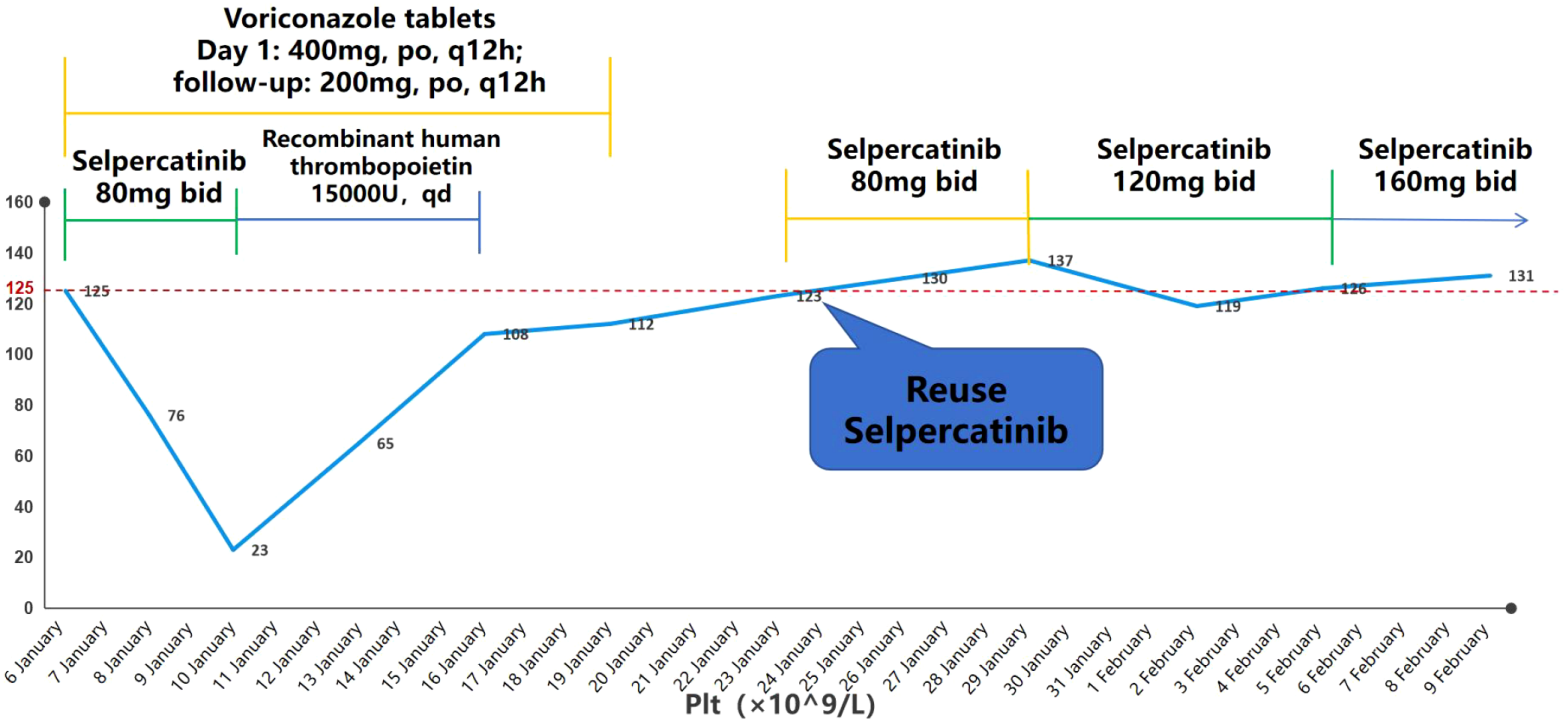
Change in PLT during the patient’s hospitalization.

The main medications used by the patient during hospitalization are shown in **Table 1**, and the changes in PLT are shown in **Figure 1**.

**Table 1:** Main medications used during the patient’s hospitalization.

Name of drug	Dosage	Starting and ending date of medication
Selpercatinib capsule	160 mg, po, bid	15 November 2024 to 5 January 2025
	80 mg, po, bid	6 January 2025 to 10 January 2025
Voriconazole tablets	Day 1: 400 mg, po, q12h; follow-up: 200 mg, po, q12h	6 January 2025 to 19 January 2025
Recombinant human thrombopoietin injection	300 μg, ih, qd	10 January 2025 to 16 January 2025
Selpercatinib capsules	80 mg, po, bid	23 January 2025 to 29 January 2025
Selpercatinib capsules	120 mg, po, bid	30 January 2025 to 5 February 2025
Selpercatinib capsules	160 mg, po, bid	February 2025 to present

## 3 Discussion

Selpercatinib, launched in the United States on 8 May 2020, is the world’s first highly selective RET tyrosine kinase inhibitor approved for clinical use (1, 2). It has been approved in China for the treatment of adult patients with locally advanced or metastatic NSCLC harboring positive RET gene. Selpercatinib significantly improves progression-free survival, reduces the risk of disease progression and death in patients with RET gene fusion-positive NSCLC, and shows promise in preventing brain metastasis (3, 4). Herein, we present a case of a RET gene fusion-positive patient with advanced NSCLC who experienced an adverse reaction during concomitant administration of selpercatinib and voriconazole.

The temporal sequence of events strongly supports a drug–drug interaction. The patient maintained a normal platelet count during initial selpercatinib monotherapy. Despite a preemptive 50% dose reduction of selpercatinib within 4 days (6 January to 10 January) of adding voriconazole, he developed Grade IV thrombocytopenia. However, thrombocytopenia is a rare adverse effect of voriconazole, and the patient’s platelet count did not change significantly between 16 January and 19 January when the patient was using voriconazole alone; therefore, the patient’s thrombocytopenia was not related to the use of voriconazole alone. However, thrombocytopenia is a common adverse effect of selpercatinib, occurring in 33% of patients in the LIBRETTO-001 study of selpercatinib, including 2.7% in Grades III to IV (5). Crucially, thrombocytopenia did not recur when selpercatinib was later restarted and titrated to the full dose after voriconazole had been discontinued. This pattern of positive de-challenge and re-challenge is highly suggestive of an interaction precipitated by voriconazole. The most plausible mechanism is voriconazole-mediated inhibition of CYP3A4, the primary metabolizing enzyme for selpercatinib (2).

This leads to increased systemic exposure to selpercatinib, exacerbating its dose-dependent hematologic toxicities. Furthermore, it is important to consider other pharmacokinetic pathways to isolate the primary mechanism. Selpercatinib is known to be a substrate of P-glycoprotein (P-gp) (2). Selpercatinib exposure is restricted by P-gp efflux and predominantly metabolized by CYP3A, influencing its systemic levels (6). However, voriconazole is not a significant inhibitor of intestinal P-gp (7), making this pathway less likely to contribute substantially to the observed interaction. Additionally, while voriconazole is a potent inhibitor of CYP2C9 and CYP2C19 (8, 9), selpercatinib is not a major substrate for these isoenzymes (2, 10). Therefore, the inhibition of CYP2C9/19 by voriconazole is unlikely to have played a significant role. This analysis strengthens the conclusion that the marked increase in selpercatinib exposure—and subsequent toxicity—is attributable predominantly to CYP3A4 inhibition.

The potential role of pharmacogenetics warrants consideration. Genetic polymorphisms in CYP3A4 and CYP3A5 genes can result in significant inter-individual variability in enzyme activity (11). Voriconazole shows time-dependent inhibition of CYP3A4 and significant inhibitory effects on CYP2C19, contributing to drug–drug interactions with CYP3A4 substrates (12). Although we did not perform genetic testing in this case, it is conceivable that patients with inherently reduced CYP3A4 metabolic capacity (e.g., poor metabolizers) might be at an even higher risk for severe interactions when selpercatinib is combined with a strong inhibitor like voriconazole. This underscores the importance of heightened vigilance and perhaps more aggressive dose adjustments in populations where such polymorphisms are prevalent.

Since tyrosine kinase inhibitors (TKIs) have a broad kinase target spectrum, most TKIs inevitably have off-target effects. Platelet function is dependent on the activity of tyrosine kinases such as Tec, Btk, SFK, Lyn, Fyn, and Syk, which have been shown to be inhibited by TKIs. For example, imatinib inhibits the platelet-derived growth factor receptor (PDGFR) present in platelets, thereby blocking its downstream PI3K/Atk and MAPK/Erk signaling pathway, promoting megakaryocyte apoptosis and ultimately leading to thrombocytopenia (13). Dasatinib causes thrombocytopenia and dysfunction by inhibiting SFK, plcg2, and PI3K signaling pathways. As a multi-target TKI that also inhibits RET tyrosine kinase activity as well as selpercatinib, sunitinib, on the other hand, blocks VEGFR, PDGFR, c-kit, CSF-1R, FLT3, and RET kinases and has been shown to act on RET tyrosine kinases on platelets, manifesting with a hemorrhagic response (14). Inspired by the mechanism of action of other TKIs, the mechanism by which selpercatinib leads to thrombocytopenia is likely to be that its off-target effect allows for the inhibition of RET tyrosine kinase activity on platelets, which inhibits platelet function and in turn leads to its reduction.

Studies have shown that selpercatinib provides relatively good clinical benefit in terms of objective remission and disease control rates in the target population, but its adverse effects are common and should not be ignored (15). Therefore, during the treatment of selpercatinib, clinical pharmacists need to provide strict pharmacological supervision to patients and guide them in the rational use of medication. Grade IV thrombocytopenia occurred in this patient 4 days after concomitant treatment with selpercatinib and voriconazole, and the relevant literature (2) reported that during the course of selpercatinib medication, 42% of patients suspended treatment due to adverse drug reactions, and 5% needed to permanently discontinue selpercatinib treatment due to adverse reactions, which mainly manifested themselves in the form of thrombocytopenia and pneumonitis, among others.

In terms of drug interactions, following selpercatinib coadministration with multiple doses of the strong CYP3A inhibitor itraconazole, the AUC0-INF and *C*_max_ of selpercatinib were increased by 133% and 30%, respectively. Coadministration of selpercatinib with multiple doses of the moderate CYP3A inhibitors diltiazem, fluconazole, or verapamil is predicted to increase selpercatinib AUC (by 60%–99%) and *C*_max_ (by 46%–76%) (10). Considering that voriconazole is also a potent CYP3A4 inhibitor, the dose of selpercatinib was adjusted downward when voriconazole therapy was initiated, but the patient continued to experience thrombocytopenia, and the patient’s platelet counts returned to normal after the use of recombinant human thrombopoietin. After discontinuing voriconazole, the physician intends to restart treatment with selpercatinib, which, according to the selpercatinib drug insert, has been associated with adverse reactions in combination with a potent CYP3A4 inhibitor, and treatment with selpercatinib should be resumed only after the CYP3A4 inhibitor has been discontinued for a period of 3 to 5 half-lives. The half-life of voriconazole is 3 h (16). After discussion with the clinical pharmacist, the physician decided to restart treatment with selpercatinib at a dose of 80 mg bid 4 days after voriconazole was discontinued, and the patient’s platelet counts were normal; the patient’s platelet counts and other possible adverse reactions were closely monitored. After 7 days of treatment with 80 mg bid of selpercatinib, the patient did not have any adverse reactions, and the dose of 120 mg bid of selpercatinib was raised and treatment continued for 7 days without any adverse reactions; thus, the dose of selpercatinib was raised again to the patient’s initial dose of 160 mg bid, and the patient did not have any further thrombocytopenia during the subsequent treatment.

In conclusion, this case provides strong clinical evidence for a significant interaction between voriconazole and selpercatinib, mediated primarily by CYP3A4 inhibition. Clinicians should avoid the concomitant use of strong CYP3A4 inhibitors during selpercatinib therapy. If coadministration is unavoidable, aggressive dose reduction of selpercatinib, enhanced therapeutic drug monitoring, and vigilant hematologic surveillance are highly recommended. In addition, selpercatinib may also have drug–drug interactions with other CYP3A inhibitors such as itraconazole, CYP3A inducers such as rifampicin, CYP3A substrates such as midazolam, and CYP2C8 substrates such as repaglinide (2); therefore, selpercatinib should be carefully coadministered with the above drugs with enhanced monitoring.

## Data Availability

The original contributions presented in the study are included in the article/supplementary material. Further inquiries can be directed to the corresponding authors.
